# The pH‐dependent photophysical and spectral properties of pH‐sensing green fluorescent proteins

**DOI:** 10.14814/phy2.70625

**Published:** 2025-10-18

**Authors:** Ian M. Thornell

**Affiliations:** ^1^ Department of Internal Medicine and Pappajohn Biomedical Institute, Roy J. and Lucille A. Carver College of Medicine University of Iowa Iowa City Iowa USA

**Keywords:** acid‐base, fluorescent proteins, GFP, pHluorin

## Abstract

Protonation and deprotonation reactions significantly influence biological function, with buffers and transporters maintaining stable pH levels in the cytosol and various organs. However, pH can vary considerably in organ lumens, such as the airway surface liquid (ASL), which serves as a defense barrier for airways. Traditional microelectrodes, while precise, face limitations prompting the use of fluorescent pH sensors. Although many green fluorescent protein (GFP)‐based pH sensors have been reported, data are usually limited to their spectral properties. Photophysical properties are often reported for a single pH value. In this study, the photophysical properties of pHluorins and superfolder GFP—including a novel pH sensor pHluorin4—were compared. Purified pHluorin4 effectively reported ASL pH differences in cystic fibrosis (CF) and non‐CF epithelia. These findings provide a comprehensive summary of the photophysical and spectral properties of popular pHluorins, including the new member pHluorin4, which can be used to optimize their application in different systems.

## 1 INTRODUCTION

Protons (H^+^) readily react with biological molecules, influencing their function through protonation (gaining a proton) and deprotonation (losing a proton). In the cytosol and many organs, buffers mitigate pH fluctuations, while transporters extrude acid–base equivalents to maintain a remarkably consistent pH (Thornell et al., 2025). For example, parietal cells in the stomach maintain a stable pH despite secreting copious H^+^ into the stomach lumen because HCO^−^_3_ is removed from the cytoplasm across the basolateral membrane (Rabon & Reuben, 1990). However, while intracellular pH is similar among cells, the pH in organ lumens can vary significantly. One example is the airway surface liquid (ASL) that serves as a thin aqueous host defense barrier for airways (Abou Alaiwa et al., 2018).

Microelectrodes are precise tools for measuring pH across physiological values by sensing H^+^ activity (Haack et al., 2015; Thornell et al., 2025). Despite their accuracy, microelectrodes face limitations, including technical challenges in fabrication and application to small cells. For reporting ASL pH, investigators rarely use microelectrodes due to several practical limitations: they are challenging to fabricate, demand specialized equipment, and are difficult to integrate into environmental enclosures designed to preserve ASL hydration and gas composition—conditions essential for physiologically relevant measurements. Fluorescent pH sensors circumvent these issues. Ideally, the fluorescence of these sensors should transition between low and very bright with a pK_a_ value close to the expected steady‐state pH of the studied system, thereby maximizing sensitivity to pH changes in either direction. Suppose pH sensing arises from the deprotonation of a pH sensor and a consequent increase in sensor fluorescence. The sensor will exist as 10% deprotonated and 90% protonated 1 pH unit below its pK_a_ and as 90% deprotonated and 10% protonated 1 pH unit above its pK_a_. Thus, the pH values reported by fluorophores undergoing a simple titration reaction are limited to roughly ±1 pH unit from its pK_a_.

In the case described above, the change in fluorescence follows a titration curve. The magnitude of the curve will depend on the fluorophore concentration and the molar brightness of the fluorophore:

(1) $$\text{Brightness}\;\left(M^{-1}\times {\mathrm{cm}}^{-1}\right)=\varepsilon \;\left(M^{-1}\times {\mathrm{cm}}^{-1}\right)\times \Phi \;\left(\text{unitless}\right)$$

In this relationship, the extinction coefficient (ε) measures the ability of the fluorophore to absorb photons, and the quantum yield (Φ) measures the efficiency of converting absorbed photons into fluorescence. These photophysical properties depend on the excitation wavelength and vary with H^+^ activity for pH sensors. If several fluorophores with appropriate pK_a_ values are available to the experimenter, it is often advantageous to choose the fluorophore with the highest brightness.

**FIGURE 1:**
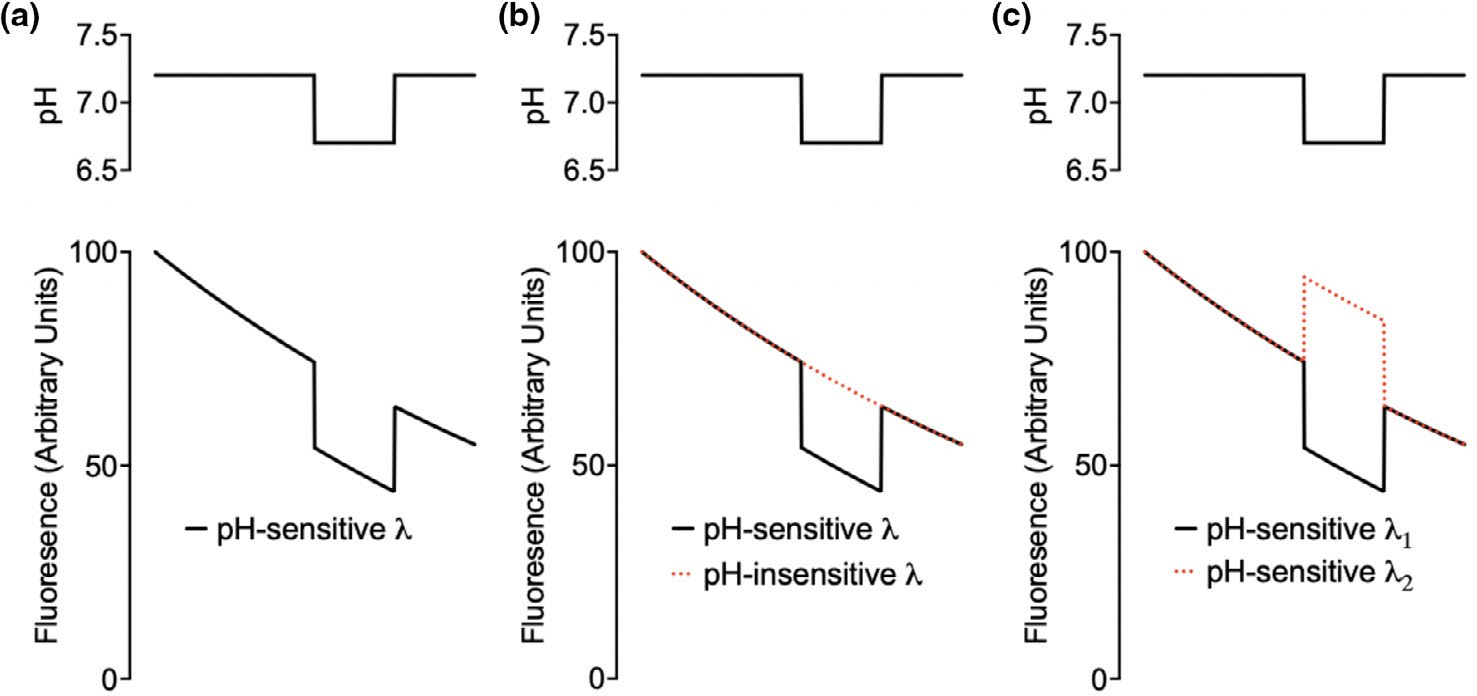
Three examples of pH sensors. Hypothetical experiments with pH‐sensing fluorophores showing the true pH value (upper trace) and hypothetical fluorescence with a decreasing signal (e.g., due to photobleaching). (a) The fluorophore emits only a pH‐dependent fluorescence but does not emit pH‐independent fluorescence. In this example, fluorescence is pH‐dependent but cannot be used to determine the true pH values. (b) The fluorophore emits a pH‐dependent and ‐independent fluorescent signal. The ratio of these signals can be used to determine the true pH values. (c) The protonated and deprotonated fluorophore emits pH‐dependent fluorescent signals with different spectra. The ratio of these signals can be used to determine the true pH values.

When using fluorescent pH sensors, it is crucial to compare the pH‐dependent fluorescence to a secondary fluorescent signal to control for variations in fluorophore concentration and any loss of fluorescent molecules during the experiment (e.g., due to photobleaching). The secondary signal can either be insensitive to pH or have the opposite relationship between pH and fluorescence compared to the primary signal (Figure 1).

Some fluorescent pH sensors are DNA‐encoded. Green fluorescent protein (GFP) and its derivatives are widely used in biological science as both protein tags and sensors. Initially, GFP's pH sensitivity was a technical limitation for its use as a tag, leading to efforts to lower its pK_a_ for stable fluorescence in mammalian cytosol. However, researchers can exploit this pH sensitivity to report pH values in cells and various biological compartments. Despite the availability of several GFP‐based pH sensors, the photophysical properties underlying their pK_a_ values are often not reported (Table 1). This study reports the photophysical properties and spectra of several previously reported GFPs for practical laser wavelengths across various pH values and compares their responses to two new GFP‐based pH sensors.

**TABLE 1:** Reported photophysical properties of GFPs.

Construct	Excitation (nm)	pK_a_	ℰ (M^−1^ cm^−1^)	Φ (unitless)	References
Enhanced GFP	489	6.15	53,000	0.60	Patterson et al. (1997) Llopis et al. (1998)
Superfolder GFP	485	6.9	49,000	0.65	Pédelacq et al. (2006) Reddington et al. (2012) Roberts et al. (2016)
Ratiometric pHluorin	396	7.1	58,000	NR	Miesenböck et al. (1998) Hess et al. (2004)
	476		38,000	NR	
pHluorin 2	405	7.1	NR	NR	Mahon (2011) Valkonen et al. (2013)
	488				
Superfolder pHluorin	390	6.9	NR	NR	Reifenrath and Boles (2018)
	470				

*Note*: The photophysical properties of several GFPs are summarized for reported excitation wavelengths. The S65T mutation in the chromophore of enhanced GFP (eGFP) and superfolder GFP (sfGFP) create GFPs that exist exclusively in the phenolate form, and the corresponding pK_a_ represents fluorescent intensity changes for the phenolate GFP. The pK_a_ of pHluorins are dominated by the transition of phenol and phenolate forms of the GFP.

Abbreviation: ℰ, extinction coefficient; NR, not reported; Φ, quantum yield.

## 2 METHODS

### 2.1 Generation of cDNAs

The cDNA sequences were synthesized by Integrated DNA Technologies (IDT) as gBlocks with *XmnI* and *NotI* restriction sites. The gBlocks were digested with restriction enzymes (R0194S and R3189S New England Biolabs), then ligated into their respective vectors using a T7 DNA Ligase Kit (M0318S New England Biolabs). All vectors were confirmed by DNA sequencing (GENEWIZ). The newly generated sfGFP‐mCRISPRed (Addgene 241869) and pHluorin4 (Addgene 241868) vectors have been deposited to Addgene.

### 2.2 Expression in A549 cells

Each studied construct was placed into pcDNA5/TO mammalian expression vectors (V103320 Gibco) using appropriate restriction sites and T7 DNA Ligase Kit (M0318S New England Biolabs). After transformation, EndoFree Plasmid Maxi Kit (12362 Qiagen) was used to obtain plasmids from ampicillin‐resistant TOP10 competent cells (C404010 Invitrogen). Then, 1 μg of DNA was transfected using X‐tremeGENE HP DNA Transfection Reagent per manufacturer's instructions (6366244001 Millipore Sigma). Images of green fluorescent proteins (GFPs) were captured using a 488‐nm excitation from a Leica CTR6000 inverted microscope equipped with a 20x Plan APO objective (0.70 NA) and a Leica DFC310 FX camera using Leica Application Suite X v3.7.3.23345 software.

### 2.3 Purification of GFPs

Each cDNA was cloned into pMAL‐5 vectors provided in the pMAL Protein Fusion & Purification Kit (E8201S New England Biolabs) that encodes the opening reading frame with a maltose binding protein. BL21(DE3) competent cells (C600003 Invitrogen) were transformed with a single GFP‐encoding pMAL‐5 plasmid. After selection, each BL21(DE3) construct was stocked in glycerol. Stocked bacteria were streaked onto ampicillin plates and incubated overnight at 37°C. The next day, 10 mL LB medium containing 1 mg ampicillin (A40040, Research Products International) was inoculated with GFP‐expressing bacteria and shaken at 37°C until a 600‐nm absorbance of 0.6–0.8 was obtained; then the bacteria were stored overnight at 4°C. The next day, 1 L pre‐autoclaved rich broth [10 g tryptone (211705 Fisher Scientific), 5 g yeast extract (210929 Fisher Scientific), 5 g NaCl (S23020 Research Products International), 2 g glucose (G32040 Research Products International), 100 mg ampicillin (A40040, Research Products International)] was inoculated with GFP‐expressing bacteria. Bacteria were grown on a shaker at 37°C to a 600‐nm absorbance of 0.6–0.8. GFP expression was induced by adding 72 mg isopropyl β‐D‐1‐thiogalactopyranoside (IPTG, I56100, Research Products International). Analysis of fluorescent intensity revealed peak expression 1.5–2 h after IPTG addition. Therefore, a 2 h time point was used to harvest GFPs. Rich broth was centrifuged at 4000 × *g* for 20 min and cells were resuspended in 25 mL pH 7.40 column buffer [per liter: 20 mL of 1 M Tris–HCl pH 7.40 (prepared from TRIS, T60040 Research Products International and HCl, 470301 VWR), 11.7 g NaCl (S23020 Research Products International), 2 mL 0.5 M EDTA (409975000 Fisher Scientific)]. Bacteria in column buffer were stored at −20°C overnight and up to 1 week.

To obtain GFPs from bacteria, frozen bacteria in column buffer were thawed in cold water. Samples were sonicated on ice for 15 s with 1 min rest for a total of 2 min of sonication (Fisher Scientific Sonicator 100, power 4). At this time, peak protein levels were reached as observed by Bradford assay (5000006 Bio‐Rad) after each sonication. Samples were pelleted by centrifugation at 20,000 × *g* for 20 min. The protein concentration of the supernatant was obtained using a Pierce BCA kit (23225 Thermo Scientific), then 30 mg of protein was diluted 1:6 with additional column buffer.

To purify GFP, 2 mL amylose resin (E8021L New England Biolabs) was added to a 2.5 × 10 cm column (Talon 2 mL, 635606 Takara) and washed with 10 mL column buffer. Then, the 30 mg sample was loaded into the column and washed with an additional 24 mL column buffer. GFPs were eluted using column buffer containing 10 mM maltose (M22000 Research Products International) to disrupt the interaction of maltose binding protein‐GFP with the amylose. Of note, the MBP‐backbone plasmid encodes a TEV (Tobacco Etch Virus) protease site that could be used if elimination of the MBP is desired.

### 2.4 Assessment of photophysical and spectral properties

All photophysical and spectral properties were acquired with a SpectraMax i3x (Molecular Devices) from a single harvest of each GFP construct. A common stock solution with all Good buffers (Good et al., 1966) was titrated to various pH values at 37°C [in mM: 110 NaCl (S23020 Research Products International), 10 MES (2‐(N‐morpholino)ethanesulfonic acid, M22040 Research Products International), 10 PIPES (piperazine‐N,N′‐bis(2‐ethanesulfonic acid), P40140 Research Products International), 10 HEPES (4‐(2‐hydroxyethyl)‐1‐piperazineethanesulfonic acid, H75030 Research Products International), 10 tricine (T24000 Research Products International)] and fluorophores resuspended to optical densities ≤0.05 to minimize the inner filter effect. All properties of GFPs were obtained for 6 pH values. Extinction coefficients were obtained by measuring absorption for a given wavelength for a serial dilution of GFP. The [GFP chromophore] was obtained by exciting the phenolate anion chromophore in 0.1 N NaOH (SS276‐4 Fisher Scientific). Under these denaturing conditions, ε = 44,100 M^−1^•cm^−1^ for all GFPs (Patterson et al., 1997). Quantum yields were obtained by collecting fluorescent emissions >520 nm and comparing the integration of that emission spectra to a fluorescein reference (Fluorescein, disodium salt, pure; 173241000 Thermo Scientific) in 1 N NaOH (SS266‐4 Fisher Scientific) with known quantum yield, Φ = 0.95 (Lakowicz, 2010).

### 2.5 Purification of MBP‐GFPs used to measure ASL pH

All MBP‐GFPs used to measure ASL pH were purified, then washed in sterile PBS with divalent cations using a 30 kDa molecular weight cutoff spin column (Amicon Ultra‐4, UFC903008 Millipore). Concentration was verified in denaturing conditions, then fluorophores were further diluted to 1 μM. To measure endotoxin levels, samples were photobleached to photoconvert phenol GFP and minimize background absorption, then tested for endotoxin using a Pierce Chromogenic Endotoxin Quant Kit (A39552S Thermo Scientific) per the manufacturer's instructions. An additional photobleached sample without any chromogenic reagent was used for background subtraction for samples, and a spiked sample was used as a positive control.

### 2.6 Measurement of ASL pH

Cultured primary human airway epithelia were obtained from the University of Iowa In Vitro Models and Cell Culture Core (Karp et al., 2002) and donor details provided (Table 2). All studies were approved by the University of Iowa Institutional Review Board. Epithelial cells were cultured on Corning 0.33‐cm^2^ 0.4‐μm polyester membrane inserts (3470 Millipore) to permit imaging by an inverted microscope. Fluorophores (5 μL) were added directly into the ASL, and ASL pH was measured the next day using a Zeiss LSM880 microscope.

**TABLE 2:** Demographics of cystic fibrosis epithelia used in this study.

Age	Sex	Genotype
54	Female	∆F508/I336K
22	Male	∆F508/1717‐1G‐A
31	Female	∆F508/∆F508
64	Male	∆F508/L1254X
24	Female	∆F508/G551D
29	Female	∆F508/3876delA

### 2.7 Statistics

Titration curves were made using GraphPad Prism software and a modified acid–base titration equation (Boyarsky et al., 1988):

(2) $$y=a+b\left(\frac{10^{\left(\mathrm{pH}-{\mathrm{pK}}_{a}\right)}}{1+10^{\left(\mathrm{pH}-{\mathrm{pK}}_{a}\right)}}\right)$$

where *y* is a measurement value reported on the *y*‐axis. Variables pH and pK_a_ have their chemical definitions. Variables *a*, *b*, and pK_a_ are obtained from fitting. Where appropriate, two‐tailed unpaired *t*‐tests were performed and a *p* value of 0.05 was used for statistical significance.

## 3 RESULTS

### 3.1 Expression of GFPs in A549 cells

**FIGURE 2:**
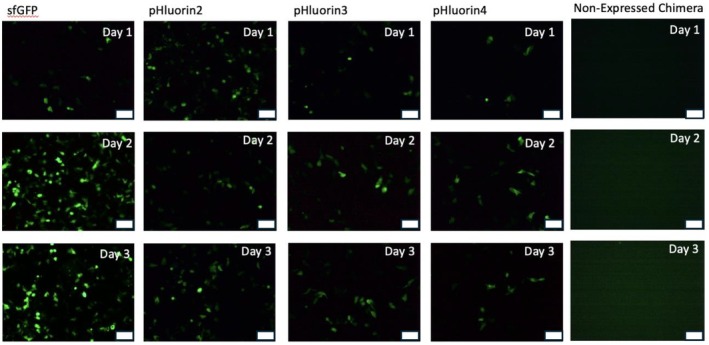
Expressing engineered GFPs in A549 cells. A549 cells were transfected with cDNA encoding different GFPs. Expression was evaluated by exciting GFPs with a 488 nm laser and collecting the fluorescent emission. The chimera that failed to express was not studied any further. Scale bar = 80 μm.

With the goal to develop novel green fluorescent proteins with enhanced pH‐sensing properties, two new green fluorescent proteins (GFPs) were engineered by combining mutations from superfolder GFP (sfGFP) and pHluorin2. These chimeras, along with the previously described sfGFP‐pHluorin2, were expressed in A549 cells, with sfGFP and pHluorin2 serving as controls (Table 3, see Appendix S1: Supplemental Sequences for complete amino acid sequences). Interestingly, while one of the novel chimeras successfully expressed in mammalian cells, the other did not (Figure 2). The expressed GFP was named pHluorin4, informally recognizing the previously reported superfolder‐pHluorin2 chimera as pHluorin3 in this report.

**TABLE 3:** Amino acids differences in pHluorins compared to sfGFP.

pHluorin2	pHluorin3	pHluorin4	Unnamed pHluorin (not expressed)
R30S			
N39Y			
**T65S**	**T65S**	**T65S**	
R80Q			
S99F			
T105N			
E132D		E132D	E132D
F145Y			
S147E	S147E	S147E	S147E
N149L	N149L	N149L	N149L
T153M			
	I161T		
A163V			
N164I	N164I	N164I	N164I
K166Q	K166Q	K166Q	K166Q
I167V	I167V	I167V	I167V
R168H	R168H	R168H	R168H
V171I			
S202H	S202H	S202H	S202H
V206A			
L220F		L220F	L220F

*Note*: Each row represents an amino acid that is different in at least one pHluorin compared to the sfGFP sequence (GenBank: ASL68970). Chromophore‐forming amino acids are in bold and filled cells (black) contain the amino acid expressed in sfGFP at that position. Complete sequences are available as Appendix S1.

### 3.2 Photophysical properties of pH‐sensitive fluorophores

**FIGURE 3:**
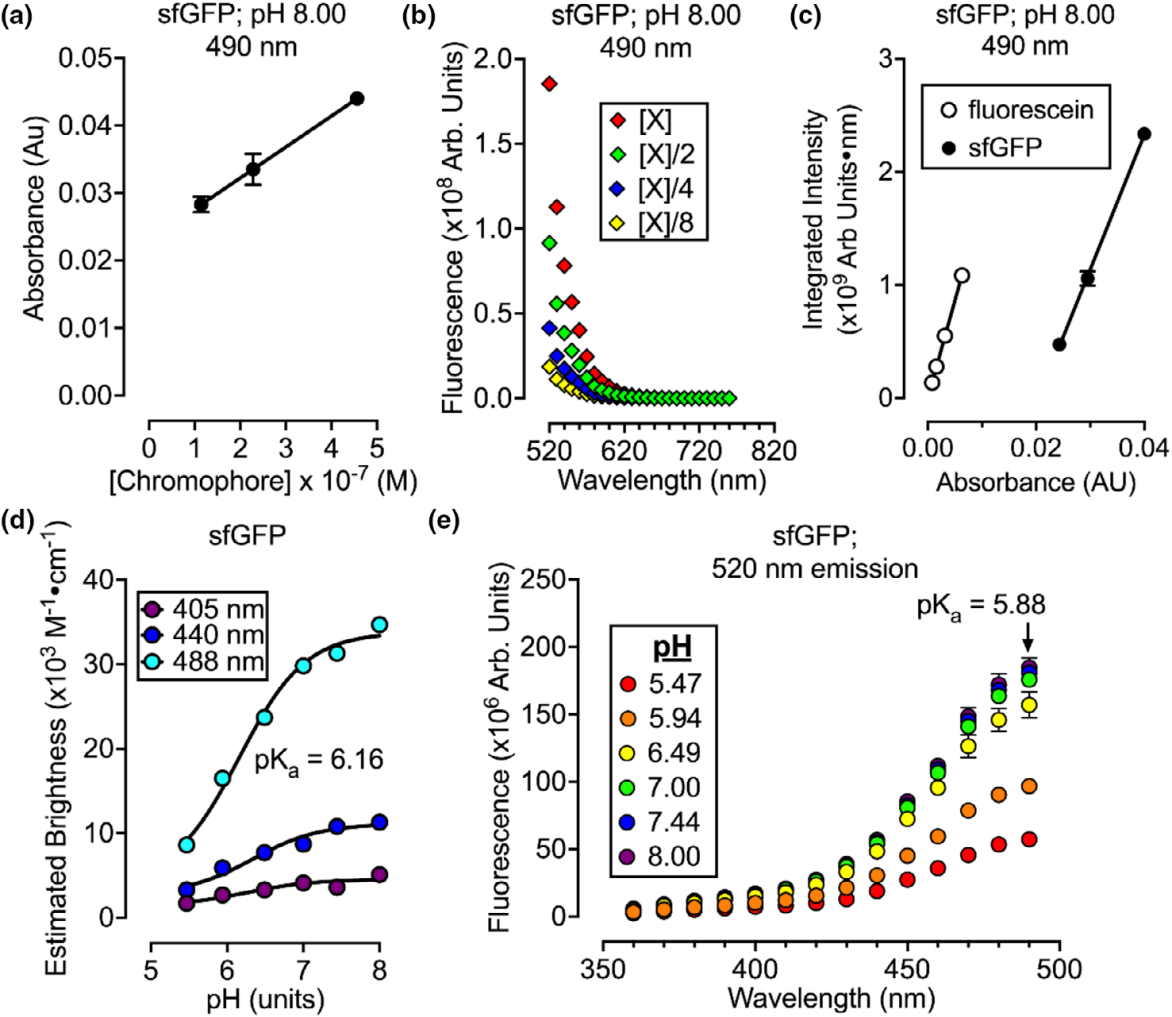
Photophysical and spectral properties of superfolder GFP. Experiments (*n* = 3) were performed on purified superfolder GFP (sfGFP). (a) Data used to calculate the extinction coefficient reported in Table 3. (b) Emission data used to obtain the quantum yield reported in Table 3. (c) For sfGFP, data are from integrating panel b data. Data from experiments with fluorescein are presented as the reference quantum yield. The ratio of these slopes was used to compute the extinction coefficient reported in Table 3. (d) Estimated brightness computed by multiplying the extinction coefficient by the quantum yield for each pH value. (e) The excitation spectra of sfGFP with a fixed 520 nm emission performed at different pH values.

Understanding the photophysical properties of pH‐sensitive fluorophores that underlie their spectra is crucial for use in biological systems. To this end, the extinction coefficients (ε) and quantum yields (Φ) of pHluorins were evaluated using a plate reader approach similar to Wall et al. (Wall et al., 2015). As a control, the photophysical properties of sfGFP were measured near conventional microscope excitation wavelengths (400 nm, 440 nm, and 490 nm). The measurement at 490‐nm excitation in pH 8.00 solutions (Figure 3a–c, and Table 4) revealed *ε* = 46,000 M^−1^•cm^−1^, closely matching the reported 49,000 M^−1^•cm^−1^ value for sfGFP in 100 mM sodium phosphate buffer, pH 8.00 (Reddington et al., 2012), and *Φ* = 0.62, similar to the reported 0.65 for sfGFP in a 10% glycerol normal saline solution, pH 7.50 (Pédelacq et al., 2006).

**TABLE 4:** Photophysical properties of sfGFP.

Measurement:	ℰ (M^−1^ cm^−1^)			Φ (unitless)			Brightness (M^−1^ cm^−1^)		
Excitation (nm):	405	440	488	405	440	488	405	440	488
pH 5.47	24,700	9900	16,600	0.07	0.33	0.52	1700	3300	8600
pH 5.94	19,000	12,300	30,000	0.14	0.48	0.55	2700	5900	16,500
pH 6.49	12,100	15,400	42,400	0.27	0.50	0.56	3300	7700	23,700
pH 7.00	10,300	13,400	48,900	0.40	0.65	0.61	4100	8700	29,800
pH 7.44	8200	16,900	54,900	0.44	0.64	0.57	3600	10,800	31,300
pH 8.00	15,000	21,700	55,900	0.34	0.52	0.62	5100	11,300	34,700

*Note*: The mean values rounded to the nearest hundred for the pH‐dependent photophysical properties of sfGFP are shown (*n* = 3 assays for each measurement).

Abbreviations: ℰ, extinction coefficient; Φ, quantum yield.

**FIGURE 4:**
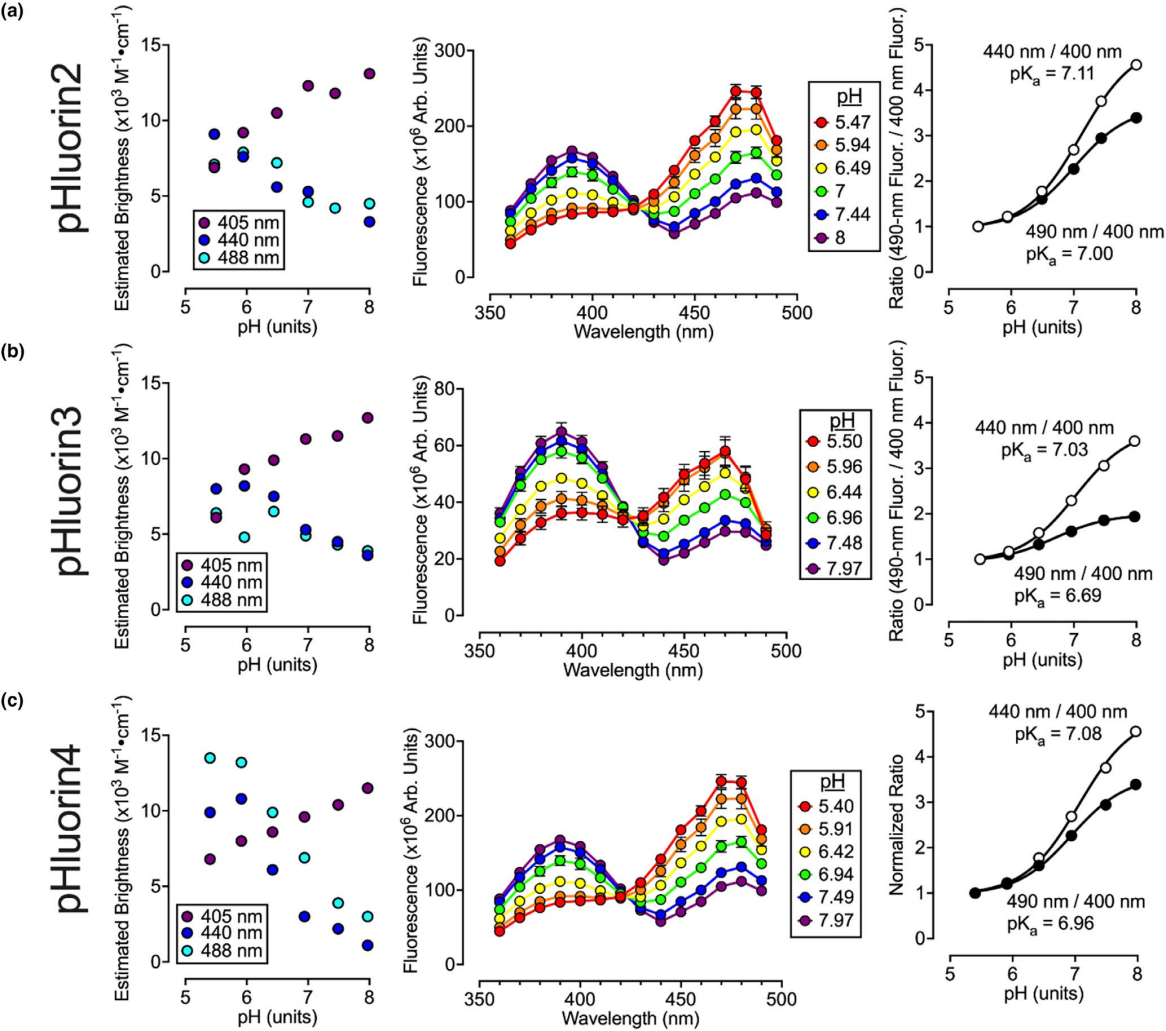
Brightness and spectra of pHluorins. Experiments (*n* = 3) were performed on purified pHluorins. (a–c) *Left* graphs report estimated brightness computed by multiplying the extinction coefficient by the quantum yield obtained for each pH value. Center graphs report the excitation spectra with a fixed 520 nm emission performed at different pH values. Right graphs report the fluorescence ratio normalized to the lowest pH values. Data were obtained for (a) pHluorin 2, (b) pHluorin 3, and (c) pHluorin 4.

Spectroscopy experiments were performed across different pH values, computing molecular brightness by multiplying each extinction coefficient by the quantum yield. This analysis revealed a pK_a_ value of 6.03 (Figure 3d). Similarly, the pK_a_ of 5.88 for fluorescence emitted with 490‐nm light (Figure 3e) was consistent with the reported pK_a_ of 5.9 for purified sfGFP fluorescence (Roberts et al., 2016). These experiments were repeated on pHluorins (Tables 5, 6, 7, Figure 4), finding that each pHluorin displayed dual excitation ranges (peaking when excited with ~400‐nm and ~490‐nm light) with each peak being less than half as bright as sfGFP. Notably, exciting the GFPs with 440‐nm light resulted in a stronger fluorescent nadir for alkaline conditions. Therefore, both excitation with 440‐nm and 490‐nm light were analyzed as possible wavelengths for ratiometric imaging with a 400‐nm excitation. Among the pHluorins, pHluorin2 and pHluorin4 exhibited the largest pH‐dependent ratiometric changes when emissions from a 440‐nm excitation were normalized to those of a 400‐nm excitation. For ASL pH measurements, pHluorin4 was selected over pHluorin2 due to its folding mutations that potentially enhance its signal through increased expression.

**TABLE 5:** Photophysical properties of pHluorin2.

Measurement:	ℰ (M^−1^ cm^−1^)			Φ (unitless)			Brightness (M^−1^ cm^−1^)		
Excitation (nm):	405	440	488	405	440	488	405	440	488
pH 5.47	8900	11,100	13,700	0.78	0.82	0.52	6900	9100	7100
pH 5.94	14,800	10,500	12,800	0.62	0.72	0.62	9200	7600	7900
pH 6.49	13,800	7500	10,700	0.76	0.75	0.67	10,500	5600	7200
pH 7.00	16,600	5900	11,500	0.74	0.9	0.4	12,300	5300	4600
pH 7.44	17,100	1900	8700	0.69		0.48	11,800		4200
pH 8.00	20,800	6700	7200	0.63	0.49	0.63	13,100	3300	4500

*Note*: The mean values rounded to the nearest hundred for the pH‐dependent photophysical properties of pHluorin2 are shown (*n* = 3 assays for each measurement). Filled cells (black) indicate that fluorescence was too near background to estimate quantum yield for λ = 440 nm (note the corresponding extinction coefficient is at its nadir).

Abbreviations: ℰ, extinction coefficient; Φ, quantum yield.

**TABLE 6:** Photophysical properties of pHluorin3.

Measurement:	ℰ (M^−1^ cm^−1^)			Φ (unitless)			Brightness (M^−1^ cm^−1^)		
Excitation (nm):	405	440	488	405	440	488	405	440	488
pH 5.50	8600	15,000	7800	0.71	0.53	0.82	6100	8000	6400
pH 5.96	11,200	13,400	7100	0.83	0.61	0.68	9300	8200	4800
pH 6.44	11,000	12,700	7000	0.9	0.59	0.93	9900	7500	6500
pH 6.96	13,600	9900	5600	0.83	0.54	0.88	11,300	5300	4900
pH 7.48	13,800	6300	5900	0.83	0.71	0.73	11,500	4500	4300
pH 7.97	15,300	5300	5300	0.83	0.68	0.74	12,700	3600	3900

*Note*: The mean values rounded to the nearest hundred for the pH‐dependent photophysical properties of pHluorin3 are shown (*n* = 3 assays for each measurement).

Abbreviations: ℰ, extinction coefficient; Φ, quantum yield.

**TABLE 7:** Photophysical properties of pHluorin4.

Measurement:	ℰ (M^−1^ cm^−1^)			Φ (unitless)			Brightness (M^−1^ cm^−1^)		
excitation (nm):	405	440	488	405	440	488	405	440	488
pH 5.40	11,900	13,500	15,900	0.57	0.73	0.85	6800	9900	13,500
pH 5.91	10,900	12,800	13,300	0.73	0.84	0.99	8000	10,800	13,200
pH 6.42	12,300	10,500	12,900	0.7	0.58	0.77	8600	6100	9900
pH 6.94	12,300	6700	11,000	0.78	0.45	0.63	9600	3000	6900
pH 7.49	13,800	6100	10,500	0.75	0.36	0.37	10,400	2200	3900
pH 7.97	14,900	4400	8000	0.77	0.25	0.37	11,500	1100	3000

*Note*: The mean values rounded to the nearest hundred for the pH‐dependent photophysical properties of pHluorin4 are shown (*n* = 3 assays for each measurement).

Abbreviations: ℰ, extinction coefficient; Φ, quantum yield.

### 3.3 Generation of sfGFP‐mCRISPRed

**FIGURE 5:**
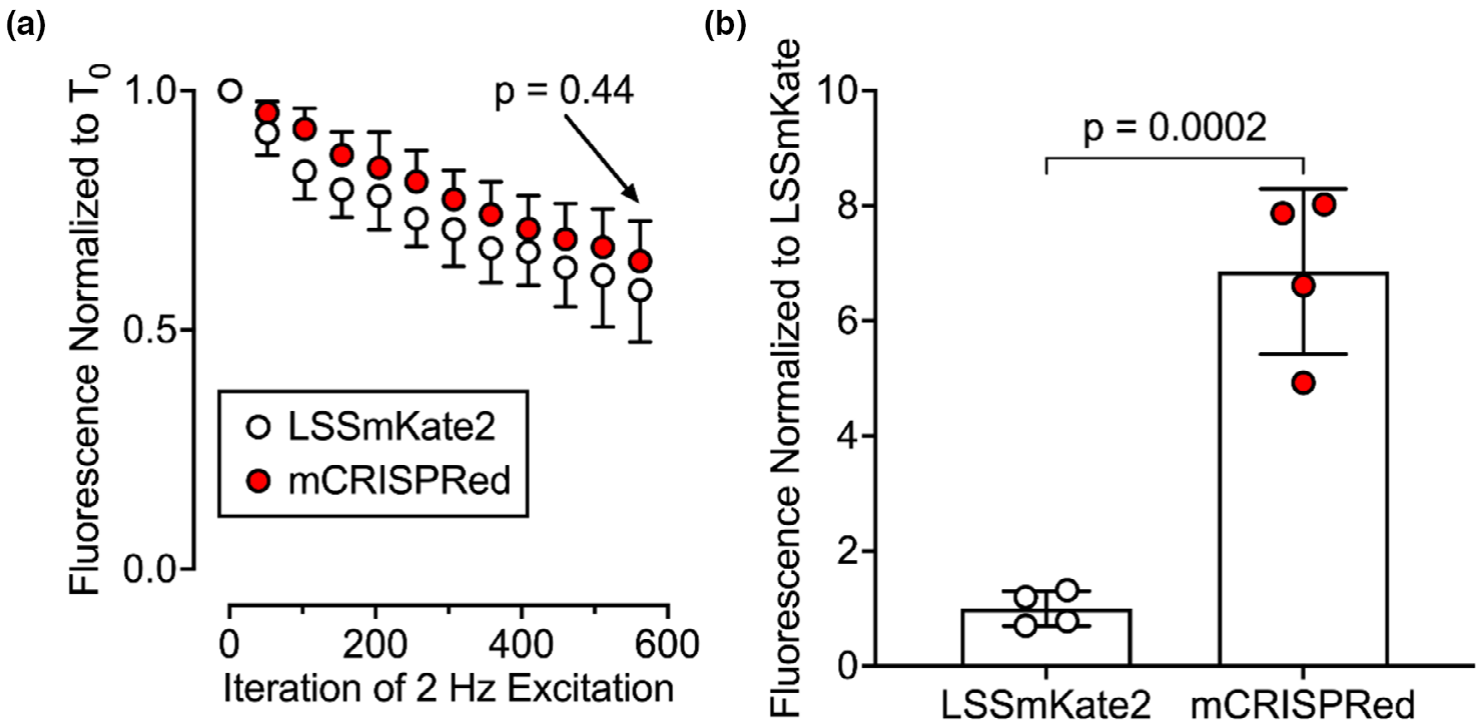
Analysis of long stokes‐shifted (LSS) fluorophores. (a) Fluorophores expressed in A549 cells were excited using 488‐nm light, and fluorescence emissions were collected over time. (b) T_0_ fluorescent intensities relative to LSSmKate2. Data were analyzed using a two‐tailed unpaired *t*‐test (*n* = 4).

As evident in the previous set of experiments, sfGFP emits bright pH‐dependent fluorescence, but lacks a second excitation‐emission pair for use as a ratiometric fluorophore. To address this, the effect of adding a second pH‐independent fluorophore that could provide an excitation‐emission pair for ratiometric imaging was tested. Two long Stokes‐shifted (LSS) fluorophores—mCRISPRed and LSSmKate2—were considered due to their low pK_a_ values and compatibility with a simultaneous 488‐nm excitation of sfGFP (peak emission ~520 nm) and the LSS‐fluorophore (peak emission ~600 nm). Despite both fluorophores having similar photobleaching properties, mCRISPRed was chosen over LSSmKate2 because of its superior brightness (Figure 5). These data are consistent with reported brightness values of 13,110 M^−1^•cm^−1^ for mCRISPRed (Erdogan et al., 2020; Subach et al., 2021) and 4420 M^−1^•cm^−1^ for LSSmKate2 (Piatkevich et al., 2010). Consequently, sfGFP was engineered in tandem with mCRISPRed.

### 3.4 Optimizing fluorophores for ASL pH measurements

**FIGURE 6:**
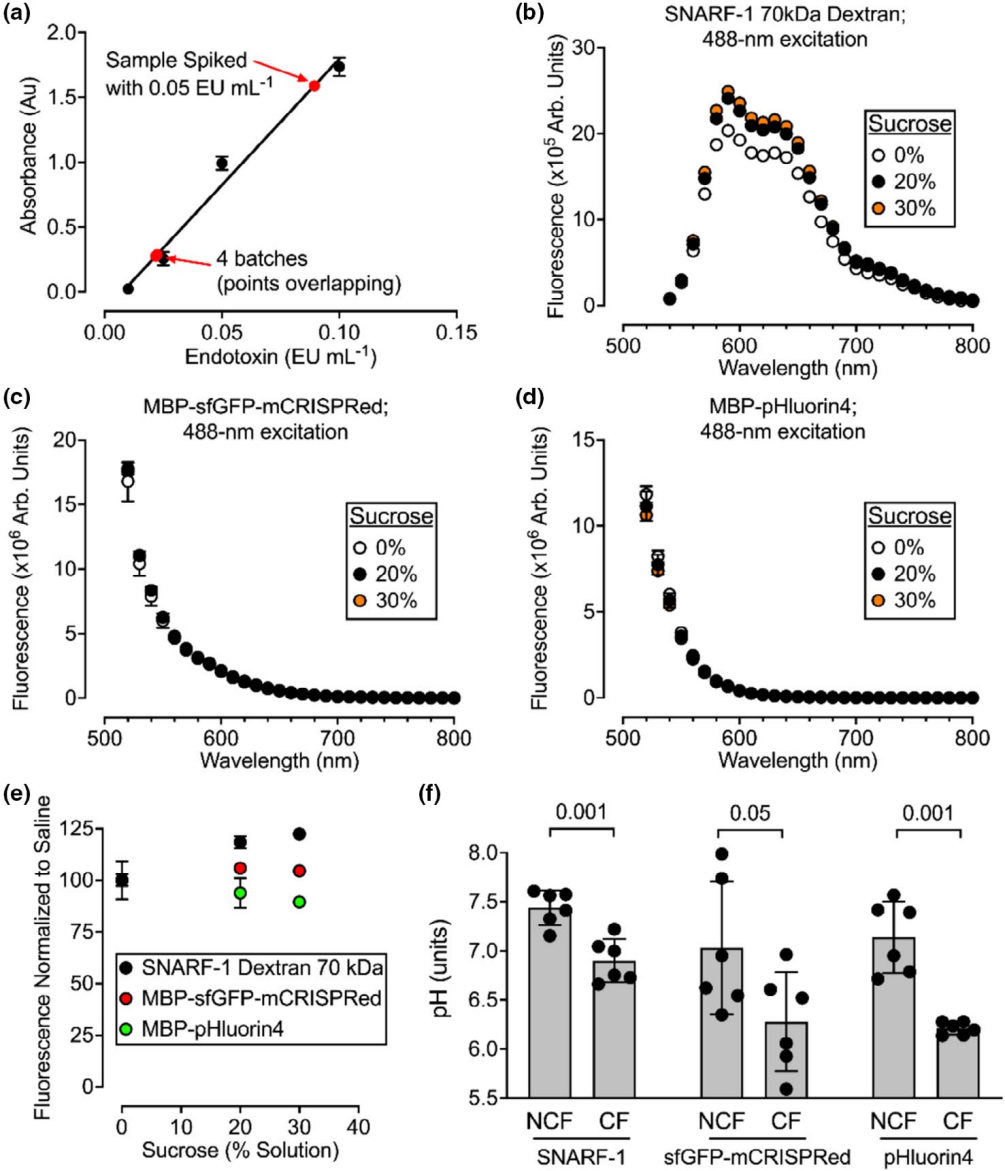
ASL pH may be reported by pHluorins. (a) Endotoxin levels in MBP‐fluorophore working solutions used to measure ASL pH (*n* = 2 purifications of MBP‐sfGFP‐mCRISPRed and *n* = 2 purifications of MBP‐pHluorin4). A pHluorin4 sample spiked with 0.05 EU mL^−1^ was included as a positive control. (b–d) Fluorescence of different indicators when viscosity is varied by sucrose addition (*n* = 3). (e) Emission spectra from panels B‐D were integrated and normalized to saline (*n* = 3). (f) The ASL pH of cultured human airway epithelia stimulated with 10 μM basolateral forskolin for 1 h. The abbreviation MBP omitted for clarity in this panel. Data were analyzed using a two‐tailed unpaired t test (*n* = 6 donors). CF, cystic fibrosis; NCF, non‐cystic fibrosis.

The next objective was to compare the precision of sfGFP‐mCRISPRed and pHluorin4 for measuring the pH of airway surface liquid (ASL). The maltose binding protein (MBP) used for purification was retained on the probes to increase the protein size and trap it in the ASL. GFP solutions contained nominal endotoxin levels (Figure 6a). Given that ASL viscosity varies and can increase in diseases like cystic fibrosis (Tang et al., 2016), the effect of viscosity on fluorescence was tested. Viscosity was altered by increasing sucrose concentrations. Using a value of 0.719 for the viscosity of water at 35°C, 20% and 30% sucrose represented relative viscosities of approximately 1.8 and 2.9 (Swindells et al., 1958). SNARF‐1 70 kDa‐dextran (D3304 Invitrogen) served as a control because it is a widely used fluorophore to monitor ASL pH. Despite changes in fluorescence with viscosity, all probes emitted >90% of their baseline fluorescence in saline (Figure 6b–e). Therefore, the effect of viscosity on reported ASL pH would be nominal, especially when constructs are used as ratiometric pH indicators.

### 3.5 Measuring airway surface liquid pH with MBP‐fusion pH sensors

Finally, 1 μM fluorophore in 5 μL phosphate‐buffered saline containing divalent cations was added to the ASL and incubated overnight. At this volume‐to‐surface area ratio, epithelia absorb excess liquid and reestablish normal ASL height by the following day (Choi et al., 2015). Similar to the stable fluorescence of rhodamines used for ASL height measurements, the bright GFP signal observed the next day indicated sufficient fluorophore stability for ratiometric pH measurements. One hour before measuring pH, fresh culture media containing 10 μM forskolin (11018 Cayman) was added to the basolateral surface of epithelia to stimulate CFTR‐dependent HCO^−^_3_ secretion, thereby enhancing the pH difference between non‐CF and CF ASL (Shah et al., 2016). Each fluorophore successfully measured differences in ASL pH between matched non‐CF and CF epithelia (Figure 6f).

## 4 DISCUSSION

In this study, the photophysical descriptions of widely used pHluorins—and newly engineered pHluorin4—were provided for a variety of wavelengths and pH values. The pK_a_ of pHluorin4 is ~7 depending on the chosen wavelengths, which is ideal for measuring cytosolic pH as well as compartments that could be mildly acidic, such as airway surface liquid. Indeed, pHluorin4 can report pH differences in non‐CF and CF ASL that occur between pH 6.00 and pH 7.50. Unexpectedly, pH measurements with sfGFP‐mCRISPRed were highly variable. Upon experimentation, the 488‐nm excitation in the mCRISPRed emission channel needed additional gain to match that of the sfGFP emission channel; therefore, it is suspected that noise in the mCRISPRed emission drove the variability in ratios, thereby pH values.

Comparing the photophysical properties of pHluorins and sfGFP provides further insights into the production of fluorescence by GFPs. GFPs can exist as a phenol excited by ~405‐nm light or a phenolate anion excited by ~488‐nm light (Patterson et al., 1997). The chromophore of both wild‐type GFP species is formed by amino acids S65, Y66, and G67 (Cody et al., 1993; Prasher et al., 1992; Zhang et al., 2006), and differences in excitation arise from the difference in the hydrogen bond networks of phenol and phenolate GFP (Chattoraj et al., 1996). The S65T variant of GFP increases the phenolate form of GFP and nearly eliminates the phenol form (Ormö et al., 1996). Consistent with these findings, the brightness of sfGFP was nominal when excited by 405‐nm light, indicating that phenolate anion sfGFP dominated in the solution. However, when excited with 488‐nm light, the S65T version of pHluorin4 did not fluoresce in mammalian cells. Based on the general finding that S65T mutations in GFPs increase emissions when excited by 488‐nm light, it is likely that the S65T pHluorin4 did not fold correctly in A549 cells.

The phenolate anion of sfGFP increased its molar brightness more than threefold with deprotonation, driven by an increase in its extinction coefficient. Interestingly, the molar brightness of the phenolate anion of pHluorins with native S65 decreased with deprotonation. Other GFPs with the native S65 can increase fluorescence with deprotonation (Ward et al., 1982). Thus, differences in the hydrogen bond network outside of the chromophore likely explain the opposite effect of pH on fluorescence for phenolate anion S65T sfGFP (or S65 wild‐type GFP) and S65 pHluorins.

The coexistence of phenol and phenolate anion pHluorins allows for dual excitation/single emission ratiometric pH imaging. The brightness increased for the phenol GFP to a similar degree among studied pHluorins, suggesting that the excited‐state proton transfer pathway (Chattoraj et al., 1996; Shu et al., 2007) is similar among these pHluorin variants, and differences in pH sensing arose from the behavior of the phenolate anion pHluorin.

Under acidic conditions, the phenolate anion pHluorin4 had increased molar brightness compared to that of phenolate anion pHluorin3, driven by a higher extinction coefficient. Based on the amino acid sequences of pHluorin3 and pHluorin4, it is likely that amino acids 132, 161, and 220 could play a role in maintaining a favorable hydrogen bond network for absorbing 488‐nm light at low pH values. This knowledge could be important for optimizing phenolate anion fluorescence under acidic conditions, such as in vesicles.

For assessing ASL pH values, two approaches were tested: using pHluorin4 and using phenolate‐dominant sfGFP in conjunction with an additional pH‐independent fluorophore, mCRISPRed. Both MBP‐conjugated pHluorin4 (~69.5 kDa) and the sfGFP‐mCRISPRed (~97.6 kDa) are expected to penetrate mucus and are capable of reporting periciliary pH (Button et al., 2012). Despite the increase in brightness of sfGFP, pH sensing was noisier compared to that of pHluorin4. One explanation may be the difference in brightness between sfGFP (46,000 M^−1^•cm^−1^) and mCRISPRed (13,110 M^−1^•cm^−1^). Increasing the per construct brightness of mCRISPRed to that of sfGFP by placing three mCRISPRed sequences in tandem could mitigate this issue. Another source of noise could be photobleaching, as each chromophore has different photobleaching kinetics. Using a single pH‐sensitive chromophore would control for photobleaching. As a note, the pH dependence of pHluorins is being driven by two distinct forms of GFP, thus the pH sensing is not arising from a single chromophore and could be prone to similar artifacts.

Although the pK_a_ value of pHluorin4 may be well‐positioned to report ASL pH compared to that of SNARF‐1, the preparation time and quality controls (e.g., measuring endotoxin) to generate pHluorin4 are a limitation. It is not expected that conjugation of pHluorin4 to MBP or other fusions would affect its pK_a_ (e.g., Miesenböck et al., 1998). However, MBP fusion can increase protein production (Bokhove et al., 2016); thus, pHluorin4 could be used without MBP fusion to minimize potential consequences of MBP fusion on cell biology.

In summary, pHluorin4 can provide accurate pH measurements from 6.0 to 7.0. An example of using pHluorin4 to report the pH of ASL was reported. While pHluorin4 was added directly to the ASL, pHluorin4 can also be genetically encoded and targeted to specific compartments based on promoters and localization signals.

## FUNDING INFORMATION

IMT was supported, in part, by the National Institute of Health (HL007638) and additional funding by the Division of Pulmonary, Critical Care, and Occupational Medicine, Internal Medicine (University of Iowa). The In Vitro Models Cell Culture Core was supported, in part, by the National Institute of Health (HL091842, HL152960) and by the Cystic Fibrosis Foundation (Iowa CFF Research Development Program).

## CONFLICT OF INTEREST STATEMENT

The author has no conflicts of interest to report.

## ETHICS STATEMENT

All studies were approved by the University of Iowa Institutional Review Board.

## Supporting information


Appendix S1.

## Data Availability

The data that support the findings of this study are available from the corresponding author upon reasonable request.
