# Correction to “The pH‐dependent photophysical and spectral properties of pH‐sensing green fluorescent proteins”

**DOI:** 10.14814/phy2.70690

**Published:** 2025-12-08

Thornell. I. M. (2025). The pH‐dependent photophysical and spectral properties of pH‐sensing green fluorescent proteins. *Physiological Reports, 13*(20), e70625.

In the published version of Figure 4, the line of text at the top of all three rightmost graphs gives 400 nm as the denominator. This was incorrect: 400 nm should be the numerator, and this was how the ratio was graphed and presented in the original Figure 4. Furthermore, the y‐axis for the two rightmost graphs in panel A and panel B should read “Normalized Ratio.” A correctly labelled version of Figure 4 is shown below.

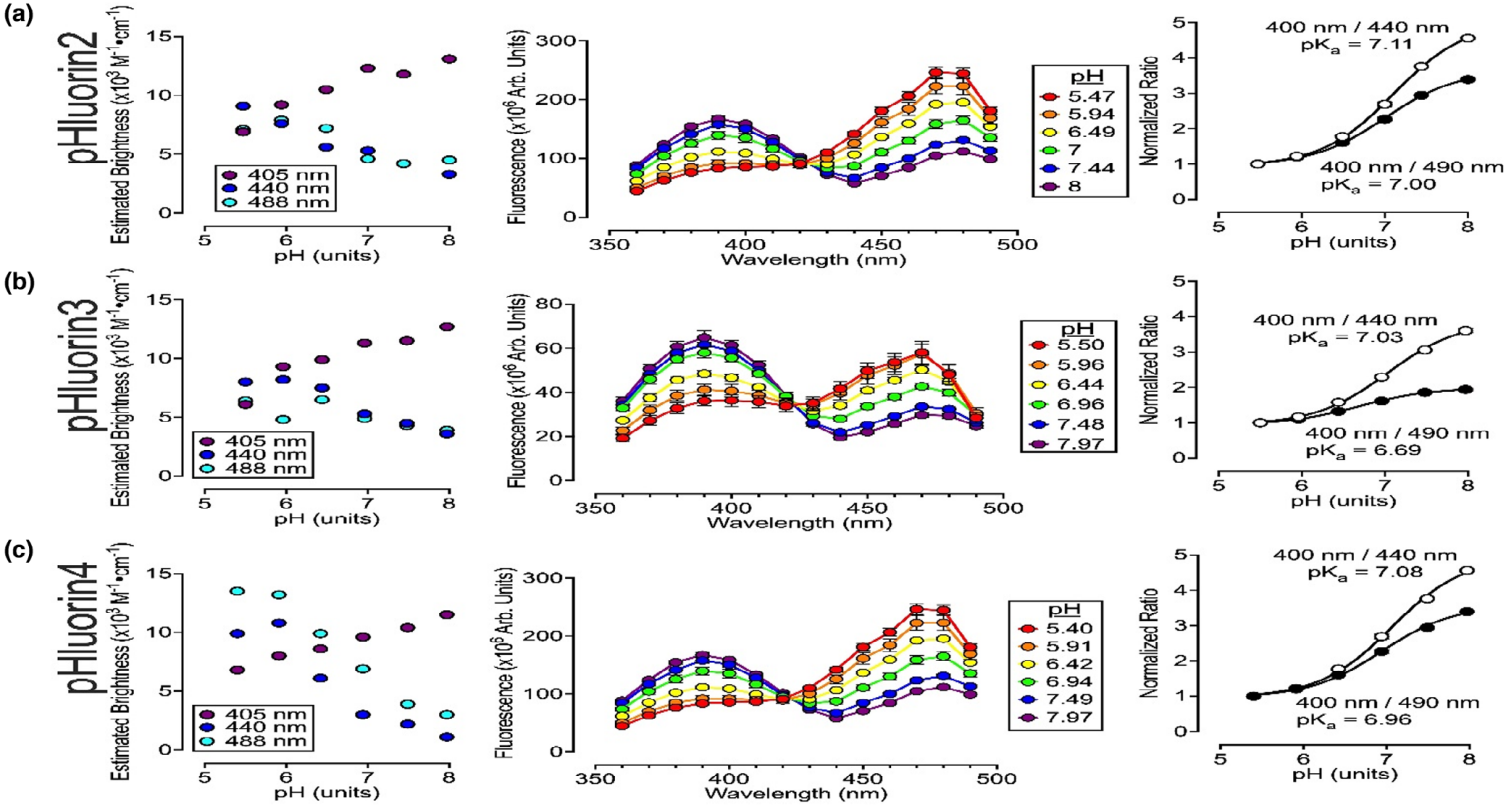

The author apologizes for these errors, which do not affect the scientific statements or conclusions in the paper.

