# Supplementary material for: The pH‐dependent photophysical and spectral properties of pH‐sensing green fluorescent proteins
Source: Physiol Rep. 2025 Oct 18;13(20):e70625. doi: 10.14814/phy2.70625 (PMC12535197; doi:10.14814/phy2.70625)
Supplement: Supplementary file 1 — Appendix S1. [file PHY2-13-e70625-s001.docx]

**Supplemental Sequences**

= difference in this amino acid position for at least 1 sequence

sfGFP

MSKGEELFTGVVPILVELDGDVNGHKFSVRGEGEGDATNGKLTLKFICTTGKLPVPWPTLVTTLTYGVQCFSRYPDHMKRHDFFKSAMPEGYVQERTISFKDDGTYKTRAEVKFEGDTLVNRIELKGIDFKEDGNILGHKLEYNFNSHNVYITADKQKNGIKANFKIRHNVEDGSVQLADHYQQNTPIGDGPVLLPDNHYLSTQSVLSKDPNEKRDHMVLLEFVTAAGITHGMDELYK

pHluorin2

MSKGEELFTGVVPILVELDGDVNGHKFSVSGEGEGDATYGKLTLKFICTTGKLPVPWPTLVTTLSYGVQCFSRYPDHMKQHDFFKSAMPEGYVQERTIFFKDDGNYKTRAEVKFEGDTLVNRIELKGIDFKDDGNILGHKLEYNYNEHLVYIMADKQKNGIKVIFQVHHNIEDGSVQLADHYQQNTPIGDGPVLLPDNHYLHTQSALSKDPNEKRDHMVFLEFVTAAGITHGMDELYK

pHluorin3

MSKGEELFTGVVPILVELDGDVNGHKFSVRGEGEGDATNGKLTLKFICTTGKLPVPWPTLVTTLSYGVQCFSRYPDHMKRHDFFKSAMPEGYVQERTISFKDDGTYKTRAEVKFEGDTLVNRIELKGIDFKEDGNILGHKLEYNFNEHLVYITADKQKNGTKAIFQVHHNVEDGSVQLADHYQQNTPIGDGPVLLPDNHYLHTQSVLSKDPNEKRDHMVLLEFVTAAGITHGMDELYK

pHluorin4

MSKGEELFTGVVPILVELDGDVNGHKFSVRGEGEGDATNGKLTLKFICTTGKLPVPWPTLVTTLSYGVQCFSRYPDHMKRHDFFKSAMPEGYVQERTISFKDDGTYKTRAEVKFEGDTLVNRIELKGIDFKEDGNILGHKLEYNFNEHLVYITADKQKNGTKAIFQVHHNVEDGSVQLADHYQQNTPIGDGPVLLPDNHYLHTQSVLSKDPNEKRDHMVLLEFVTAAGITHGMDELYK

Unnamed pHluorin (not expressed)

MSKGEELFTGVVPILVELDGDVNGHKFSVRGEGEGDATNGKLTLKFICTTGKLPVPWPTLVTTLTYGVQCFSRYPDHMKRHDFFKSAMPEGYVQERTISFKDDGTYKTRAEVKFEGDTLVNRIELKGIDFKEDGNILGHKLEYNFNEHLVYITADKQKNGTKAIFQVHHNVEDGSVQLADHYQQNTPIGDGPVLLPDNHYLHTQSVLSKDPNEKRDHMVLLEFVTAAGITHGMDELYK
